# Lung function and breathing patterns in hospitalised COVID-19 survivors: a review of post-COVID-19 Clinics

**DOI:** 10.1186/s12931-021-01834-5

**Published:** 2021-09-27

**Authors:** James A. Stockley, Eyas A. Alhuthail, Andrew M. Coney, Dhruv Parekh, Tarekegn Geberhiwot, Nandan Gautum, Shyam C. Madathil, Brendan G. Cooper

**Affiliations:** 1 Lung Function and Sleep, Outpatient Department Area 3, University Hospitals Birmingham NHS Foundation Trust, Queen Elizabeth Hospital, Mindelsohn Way, Edgbaston, Birmingham, B15 2GW UK; 2 School of Biomedical Sciences, Institute of Clinical Sciences, University of Birmingham, Birmingham, UK; 3 College of Sciences and Health Professions, Basic Sciences Department, King Saud Bin Abdulaziz University for Health Sciences, Riyadh, Saudi Arabia; 4 Critical Care, Queen Elizabeth Hospital, University Hospitals Birmingham, Birmingham, UK; 5 Birmingham Acute Care Research Group, Institute of Inflammation and Ageing, UoB, Birmingham, UK; 6 Institute of Metabolism and Systems Research, University of Birmingham, Birmingham, UK; 7 Respiratory Medicine, Queen Elizabeth Hospital, University Hospitals Birmingham, Birmingham, UK

**Keywords:** Interstitial Lung Disease, Respiratory Physiology, Ventilation, Infectious Disease, Critical Care and Emergency Medicine

## Abstract

**Introduction:**

There is relatively little published on the effects of COVID-19 on respiratory physiology, particularly breathing patterns. We sought to determine if there were lasting detrimental effect following hospital discharge and if these related to the severity of COVID-19.

**Methods:**

We reviewed lung function and breathing patterns in COVID-19 survivors > 3 months after discharge, comparing patients who had been admitted to the intensive therapy unit (ITU) (n = 47) to those who just received ward treatments (n = 45). Lung function included spirometry and gas transfer and breathing patterns were measured with structured light plethysmography. Continuous data were compared with an independent t-test or Mann Whitney-U test (depending on distribution) and nominal data were compared using a Fisher’s exact test (for 2 categories in 2 groups) or a chi-squared test (for > 2 categories in 2 groups). A p-value of < 0.05 was taken to be statistically significant.

**Results:**

We found evidence of pulmonary restriction (reduced vital capacity and/or alveolar volume) in 65.4% of all patients. 36.1% of all patients has a reduced transfer factor (TL_CO_) but the majority of these (78.1%) had a preserved/increased transfer coefficient (K_CO_), suggesting an extrapulmonary cause. There were no major differences between ITU and ward lung function, although K_CO_ alone was higher in the ITU patients (p = 0.03). This could be explained partly by obesity, respiratory muscle fatigue, localised microvascular changes, or haemosiderosis from lung damage. Abnormal breathing patterns were observed in 18.8% of subjects, although no consistent pattern of breathing pattern abnormalities was evident.

**Conclusions:**

An “extrapulmonary restrictive” like pattern appears to be a common phenomenon in previously admitted COVID-19 survivors. Whilst the cause of this is not clear, the effects seem to be similar on patients whether or not they received mechanical ventilation or had ward based respiratory support/supplemental oxygen.

## Introduction

The severe acute respiratory syndrome Coronavirus 2 (SARS-CoV-2), which emerged from Wuhan, China in December 2019 has developed into the COVID-19 global pandemic [1]. It has become one of the most studied infections in recent medical history with a plethora of publications on pathology, immunology, physiology and virology in multiple areas of clinical specialisation. There is relatively little information published on lung function in survivors of moderate to severe disease. Furthermore, we had observed acutely that many COVID-19 survivors seem to display acute dysfunctional breathing patterns during and after their infection.

COVID-19, whilst being a multi-organ infection, is characterised primarily as a respiratory infection that often leads to pneumonia and significant pulmonary vascular complications. Like other pneumonias (SARS, MERS), early reports show that COVID-19 produces a sustained restrictive lung function pattern after survivors are discharged from hospital (at least 3 months post-hospital discharge) when reviewed in COVID-19 follow up clinics [2–5]. However, to date no published papers have reported looking for the assessment of dysfunctional breathing in COVID-19 survivors. Nevertheless, much work is now focussing on the treatment of post-COVID-19 symptoms including tackling dyspnoea, fatigue, and dysfunctional breathing [6].

We sought to determine if pulmonary restriction was evident in patients admitted with COVID-19 to the University Hospitals Birmingham NHS Foundation Trust and, importantly, whether the pattern of restriction was still evident 3 months after discharge and whether it was more consistent with interstitial damage or an extrapulmonary cause. In addition, we wanted to explore the possibility of dysfunctional breathing in these patients (using a novel technique).

We also hypothesised that hospitalised patients who had COVID-19 and attended ITU would have worse lung function than those who had been admitted with COVID-19 to the ward only. Therefore, we compared all physiological outcomes between these two cohorts.

We reviewed lung function in sequential Post-COVID-19 Review Clinic patients who had been hospitalised and qualified for our post-COVID-19 Review Clinic. These included patients who had been admitted to hospital, to a secondary care ward with or without an ITU admission and required a FiO_2_ > 40%.

We compared routine lung function (spirometry and gas transfer mainly) and breathing pattern assessment assessed by structured light plethysmography (SLP) in those who were admitted to wards only with those who spent time mechanically ventilated on ITU.

## Methods

Routine lung function testing was performed at our lung function department using MedGraphics Ultima equipment (*MGC UK, Gloucester, UK)* to recognised testing standards and quality control [7–11] and included spirometry and gas transfer in most patients, with a few also having lung volume estimation by nitrogen washout. As this was data from routine clinics, not all patients did every test (see “Results”) because they had other extensive assessments at these clinic appointments. Spirometry parameters included FEV_1_, FVC, FEV_1_/FVC, VC and PEF, whilst single breath carbon monoxide gas transfer test measured gas transfer (TL_CO_), transfer coefficient (K_CO_) (both corrected for haemoglobin concentration) and alveolar volume (V_A_). V_A_ in the absence of any airflow obstruction was used as a surrogate for total lung capacity (TLC). However, a few patients in each group were able to perform measurements of TLC when time allowed in this busy clinic.

Reference ranges included Global Lung Initiative (GLI) [12] for spirometry and European Coal and Steel Community (ECSC) [13] for gas transfer and lung volumes. All data (Table 2) were presented as absolute values, percent of predicted (%predicted) and as standardised residuals (SR), with SR values < −1.64 SR deemed to be below the normal range and values > 1.64 SR above the normal range [8].

Patients also had their breathing patterns assessed using the Thora 3Di Structured Light Plethysmography (SLP) device (*Pneumacare, Ely, UK*), which is an opto-plethysmographic, non-invasive measurement of chest wall and abdominal motion [14, 15]. Parameters of interest derived from SLP include relative thoracic contribution (RTC), duty cycle (ratio of inspiratory time to total breath time; Ti:Ttot), abdominal-thoracic phase angle (PA), respiratory rate (RR), and the inspiratory:expiratory flow ratio at 50% tidal volume (IE50). The variation (entropy) of breathing was also calculated from the standard deviation of breath to breath (SDBB) interval and the root mean square of the successive differences between each breath (RMSSD). Values were compared with novel reference ranges [16].

Patients who tested positive for COVID-19 and had moderate to severe symptoms required supplemental oxygen of > 40% were included in the clinics [17]. All patients who remained on wards were not ventilated (neither NIV nor nasal CPAP), but all patients admitted to ITU received mechanical ventilation requiring intubation and sedation. The only exclusion to the review was the inability to perform acceptable lung function tests.

All statistical analyses were performed using IMB® SPSS® Statistics Version 24 (Portsmouth, UK). Data distribution was initially assessed by a Shapiro–Wilk test and confirmed by visual analysis of the stem-leaf plots. Continuous data were compared between groups using either an independent t-test or a Mann Whitney-U test, depending on the distribution. Nominal data were compared between groups using either a Fisher’s exact test (for 2 categories in 2 groups) or a chi-squared test (for > 2 categories in 2 groups). A p-value of < 0.05 was taken to be statistically significant for all analyses.

## Results

### Subjects

64 male and 28 female COVID-19 survivors had their lung function measured. 45 had been treated on wards and 47 were admitted to ITU. More males with COVID required hospitalisation, with 38 (80.9%) males being admitted to ITU compared with 26 (57.8%) treated on wards (p = 0.04). Only 3 patients were current smokers, 28 ex-smokers with the rest having never smoked significantly. There were no differences in smoking status between the ITU and ward patients. Body habitus showed 65.2% obese, 28.3% overweight, 5.4% within the normal range and 1.1% underweight. There were no statistical differences in BMI between the two groups. Whilst there were more Asian patients on ITU and fewer black patients, overall the ethnicities were not statistically different. There were similar numbers of Caucasian patients in both groups. The mean (SD) duration of admission for ITU and ward patients was 40.3 (16.6) versus 9.2 (5.8) days, respectively. Underlying chronic conditions such as diabetes, respiratory and cardiac disorders have not been identified. Table 1 summarises the anthropometric data (age, sex, weight and BMI), ethnicity and clinical data.

**Table 1:** Anthropometric and clinical data for COVID-19 survivors

	Ward	ITU	All
N =	45	47	92
Male: Female	26 M: 19F	38 M: 9F*	64 M: 28F
Age (years)	54.7 (24.0–83.0)	55.5 (21.0–77.0)	56.0 (21.0–83.0)
Weight (kg)	87.0 (78.0–105.0)	93.0 (86.5–107.9)	91.5 (79.8–106.2)
BMI (kg/m^2^)	30.7 (28.2–35.6)	32.2 (29.8–37.1)	31.8 (28.7–35.89)
Underweight	1 (2.1%)	0 (0.0%)	1 (1.1%)
Normal	3 (6.7%)	6 (12.8%)	10 (10.8%)
Overweight	16 (35.6%)	10 (21.3%)	27 (29.3%)
Obese	25 (55.5%)	31 (66.0%)	54 (58.7%)
Ethnicity			
Asian	14 (31.1%)	21 (44.7%)	35 (38.0%)
Black	5 (11.1%)	1 (2.1%)	6 (6.5%)
Caucasian	24 (53.3%)	24 (51.1%)	48 (52.2%)
SE Asian	2 (4.4%)	1 (2.1%)	3 (3.3%)
Smoking history			
Current	2 (4.4%)	1 (2.1%)	3 (3.3%)
Ex	14 (31.1%)	13 (27.7%)	28 (30.4%)
Never	29 (64.4%)	31 (70.2%)	52 (64.1%)
Haemoglobin (g/L)	129.6 (2.7)	136.0 (2.2)	133.0 (1.8)
< 120 g/L	7 (14.9%)	12 (25.5%)	19 (20.2%)
Admission Details (days)			
Ward Duration	9.2 (5.8)	13.6 (8.8)	–
ITU Duration	n/a	26.3 (9.2)	–
In-Patient Stay	9.2 (5.8)	40.3 (16.6)	–

Patients include those treated on medical wards only (Ward), those that attended intensive therapy unit (ITU) and the total group (ALL). Age is presented as Mean (range), Admission Details are presented as Mean (SD), and all other data are presented as Mean (SE), Median (IQR), or number (% cohort). The only significant difference was the proportion of males to females, where there was a higher proportion of males on ITU versus Ward (*p = 0.02)

**Fig. 1:**
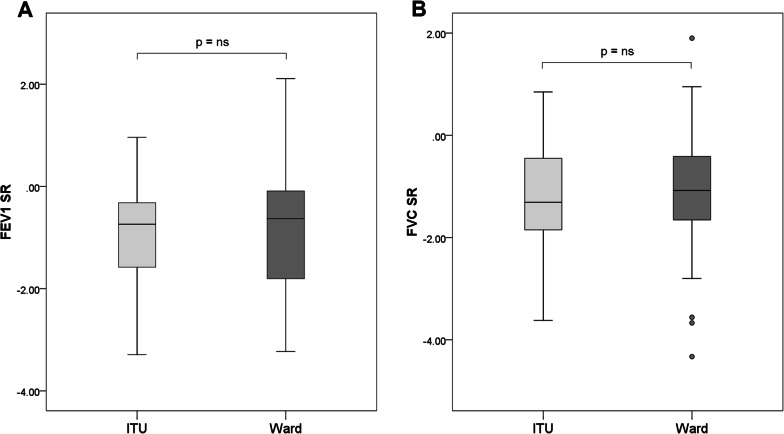
Box and whisker plots comparing FEV_1_ SR (**A**) and FVC SR (**B**) in COVID-19 survivors. Neither comparison between ward and ITU patients was statistically different. Patients include those who were treated on ITU (light grey boxes, n = 42) and those who were treated on the ward alone (dark grey boxes, n = 45). Boxes represent the IQR with the median line displayed. Whiskers represent 1.5xIQR to highlight outliers

All lung function data are summarised in Table 2. There were no differences in spirometry between the ward and ITU patients (Fig. 1). Interestingly, only 2 patients showed evidence of peripheral airflow obstruction (FEV_1_/FVC < −1.64 SR) and none showed and evidence of upper airway obstruction (defined as an Empey index > 10).

**Table 2:** Lung function data summary for COVID-19 survivors

	WARD				ITU				ALL			
	Absolute	% Predicted	SR	% Abnormal	Absolute	% Predicted	SR	% Abnormal	Absolute	% Predicted	**SR**	**% Abnormal**
Spirometry	N = 45				N = 42				N = 87			
FEV_1_	2.76 (0.11)	88.6 (2.6)	−0.73 (0.18)	23	2.81 (0.12)	85.5 (2.7)	−1.02 (0.20)	26	2.78 (0.08)	87.1 (1.86)	−0.87 (0.13)	23
FVC	3.32 (0.14)	84.6 (2.4)	−1.09 (0.19)	21	3.40 (0.16)	81.3 (2.7)	−1.39 (0.21)	34	3.36 (0.10)	83.0 (1.81)	−1.24 (0.14)	26
FEV_1_/FVC %	84.7 (79.5–88.3)	106.0 (100.0–109.5)	0.74 (0.12–1.41)	2	84.8 (81.8–87.6)	107.0 (103.0–109.8)	0.94 (0.43–1.31)	1	84.7 (80.8–88.2)	107.0 (101.0–110.0)	0.86 (0.23–1.35)	2
PEF	8.48 (6.91–10.25)	113.0 (100.5–123.5)	0.90 (0.07–1.69)	6	8.99 (7.32–9.81)	110.0 (100.3–123.8)	0.71 (0.04–1.70)	4	8.67 (7.02–10.24)	112.0 (100.0–124.0)	0.85 (0.01–1.74)	5
VC	3.44 (0.13)	87.4 (2.1)	−0.78 (0.16)	13	3.45 (0.15)	82.5 (2.6)	−1.14 (0.19)	30	3.44 (0.10)	84.8 (1.7)	−0.97 (0.13)	20
Empey Index	5.67 (4.77–6.26)	–	–	0	5.42 (4.74–6.08)	–	–	0	5.49 (4.74–6.21)	–	–	0

Lung volumes	N = 8				N = 8				N = 16			
TLC	4.78 (4.45–5.41)	80.0 (73.8–82.5)	−1.70 (−2.21–−1.67	75	5.15 (4.77–5.59)	79.5 (75.8–81.5)	−1.87 (−2.14–−1.70)	75	5.07 (4.71–5.41)	80.0 (75.0–82.3)	−1.79 (−2.17–−1.67)	75
FRC	2.43 (1.99–2.82)	70.0 (65.0–79.8)	−1.52 (−2.00–−1.17)	38	2.42 (2.19–2.75)	72.0 (63.5–82.8)	−1.50 (−2.11–−1.01)	38	2.42 (2.06–2.82)	70.5 (63.5–82.0)	−1.50 (−2.11–−1.08)	38
RV	1.50 (1.32–1.81)	70.0 (61.8–76.8)	−1.51 (−1.79–−1.16)	50	1.73 (1.70–1.84)	81.5 (76.0–84.5)	−0.91 (−1.10–−0.80)	13	1.72 (1.44–1.84)	75.0 (69.3–84.5)	−1.16 (−1.68–−0.80)	25

Gas transfer	N = 45				N = 40				N = 85			
TLco	7.17 (0.25)	79.1 (1.9)	−1.40 (0.13)	40	7.58 (0.33)	81.1 (2.07)	−1.23 (0.14)	38	7.36 (0.21)	80.1 (1.4)	−1.31 (0.10)	36
Kco	1.53 (1.36–1.66)	97.0 (86.5–108.0)	−0.26 (−1.00–0.52)*	10	1.55 (1.43–1.73)	105.0 (95.8–114.0)	0.27 (−0.32–0.82)*	8	1.54 (1.42–1.71)	102.0 (90.0–112.8)	−0.05 (−0.75–0.73)	8
VA	4.62 (0.14)	80.2 (2.0)	−1.76 (0.19)	51	4.91 (0.18)	77.6 (1.9)	−2.02 (0.18)	53	4.75 (0.12)	79.0 (1.4)	−1.87 (0.13)	50

SLP	N = 45				N = 34				N = 79			
RTC	46.71 (2.06)	84.64 (3.63)	−0.64 (0.15)	11	49.88 (2.05)	92.39 (3.81)	−0.35 (0.17)	9	48.08 (1.47)	87.98 (2.66)	−0.51 (0.11)	10
IE50	1.31 (0.06)	100.4 (4.4)	0.11 (0.16)	14	1.32 (0.04)	107.1 (3.5)	0.32 (0.23)	18	1.34 (0.04)	103.8 (2.9)	0.24 (0.13)	15
Ti:Ttot	0.40 (0.01)	104.7 (3.7)	0.58 (0.36)	23	0.43 (0.01)	106.1 (1.6)	0.71 (0.19)	21	0.41 (0.01)	101.9 (2.3)	0.37 (0.22)	22
PA	4.70 (3.60–8.50)	132.0 (93.6–236.9)	0.50 (−0.09–1.64)	28	5.50 (3.70–6.75)	154.3 (97.2–191.4)	0.81 (−0.18–1.25)	14	5.10 (3.60–7.60)	138.8 (93.8–214.5)	0.73 (−0.12–1.52)	22
RR	15.95 (0.93)	111.4 (6.3)	0.53 (0.22)	21	17.71 (0.88)	119.1 (6.0)	0.57 (0.21)	21	16.70 (0.65)	112.0 (4.5)	0.39 (0.16)	19
SDBB	0.41 (0.16–0.75)	–	–	–	0.31 (0.17–0.54)	–	–	–	0.36 (0.16–0.64)	–	–	–
RMSSD	0.38 (0.17–0.86)	–	–	–	0.34 (0.18–0.61)	–	–	–	0.37 (0.18–0.72)	–	–	–

Oximetry	N = 45				N = 47				N = 92			
SpO_2_	97 (96–98)	–	–	0	97 (96–97)	–	–	0	97 (96–98)	–	–	0

Of all lung function parameters, only K_CO_ was statistically different, with a higher SR value being observed in ITU patients compared to those treated on the ward (*p = 0.03). Patients include those treated on medical wards only (WARD), those who attended intensive therapy unit (ITU) and the total group (ALL). Absolute, % predicted and SR values are displayed as either Mean (SE) or Median (IQR), depending on the distribution of data. Normality is defined as an SR value between −1.64 and 1.64

Spirometry: Forced Expiratory Volume in 1 s (FEV_1_), Forced Vital Capacity (FVC), Peak Expiratory Flow (PEF), Relaxed Vital Capacity (VC). Lung volumes: Total Lung Capacity (TLC), Residual Volume (RV) and Functional Residual Capacity (FRC); Gas transfer: Transfer Factor (TL_CO_), Transfer Coefficient (K_CO_) and Alveolar Volume (V_A_) for single breath carbon monoxide test. All volumes are expressed in litres corrected for body temperature and pressure saturated with water (BTPS). TL_CO_ is expressed in (mmol/kPa/min) and K_CO_ in (mmol/kPa/min/L). Empey Index is FEV_1_/PEF in (mL/L/min). SLP values include; Relative Thoracic Contribution (RTC) expressed as a percentage (%), Inspiratory:Expiratory flow at 50% of tidal volume (IE50), Duty Cycle (Ti:Ttot) is the ratio of time of inspiration to total breathing cycle time, Phase Angle (PA) is expressed in degrees and Respiratory Rate (RR) is expressed in breaths per minute. The variability of breathing is expressed as the Standard Deviation of the Breath by Breath interval (SDBB) and the Root Mean Square Standard Deviation (RMSSD)

We observed evidence of pulmonary restriction (FVC and/or V_A_ < −1.64 SR) in 52 patients (55.3%), which has been previously reported in other COVID-19 studies [2–5]. In a small subgroup that performed lung volumes, reduction in TLC was confirmed but lung volumes results not different between ward and ITU patients.

**Fig. 2:**
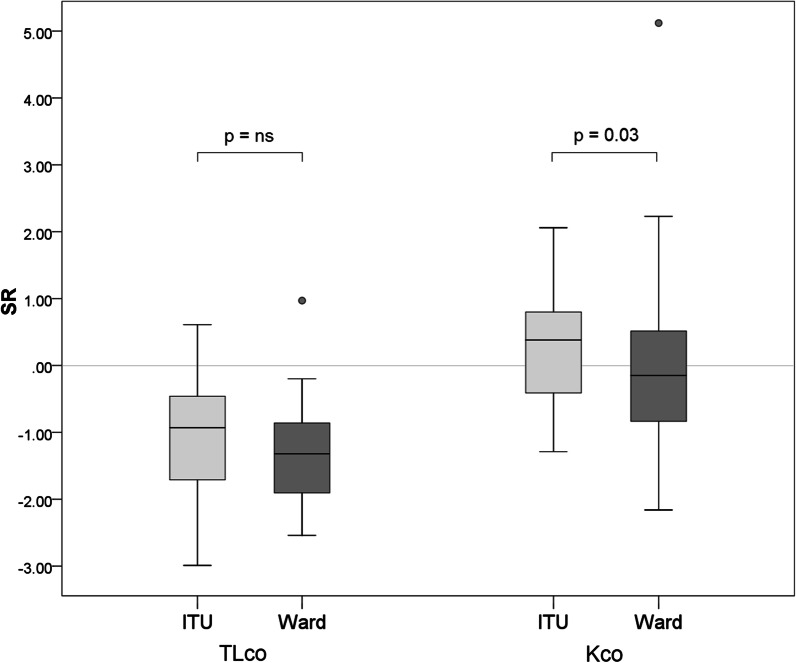
Box and whisker plots comparing TL_CO_ and K_CO_ in COVID-19 survivors. TL_CO_ and K_CO_ are displayed together to demonstrate how TL_CO_ is reduced on average, whereas K_CO_ is generally preserved (ward) or raised (ITU). Indeed, K_CO_ was significantly higher in ITU patients compared to those treated on the ward (p = 0.03). Patients include those who were treated on ITU (light grey boxes) and those who were treated on the ward alone (dark grey boxes). Boxes represent the IQR with the median line displayed. Whiskers represent 1.5 × IQR to highlight outliers

**Fig. 3:**
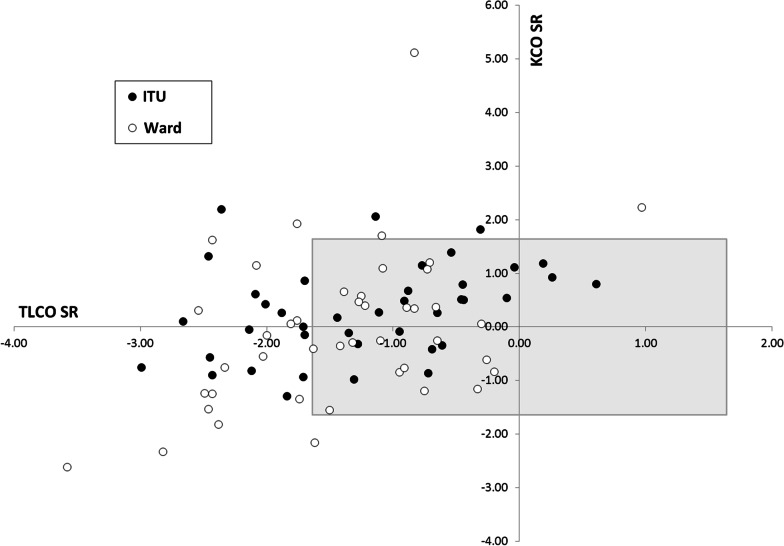
A scatter plot of TL_CO_ versus K_CO_ in COVID-19 survivors. There is a large degree of overlap between the two cohorts, although TL_CO_ is generally reduced, with 32% of all patients being below the lower limit of normal (LLN). K_CO_ was generally maintained or raised (suggesting an extrapulmonary restrictive pattern), with the latter being more common in ITU patients. Ward patients more commonly showed a reduction in both TL_CO_ and K_CO_ together (consistent with parenchymal disease) and no ITU patients had a K_CO_ below LLN. Patients include those who had been treated on ITU (closed circles, n = 40) versus those treated on the ward alone (open circles, n = 45). Data are expressed as standardised residuals (SR), with the shaded area indicating the normal range of −1.64 to 1.64 SRs for both parameters

We confirmed the reduction in gas transfer (TL_CO_) in 32 patients (34.0%) but also noted the relative preservation/increase in K_CO_ in the majority (78.1%) of these. Although TL_CO_ was not different between the two groups, K_CO_ SR was significantly higher on average in ITU patients (p = 0.03) (Fig. 2). Although there was a large degree of data overlap, there was a tendency for ward patients to show a more “parenchymal” pattern (decreases in both TL_CO_ and K_CO_ together) whereas ITU survivors showed a more “extrapulmonary” pattern (reduced TL_CO_ with a normal or raised K_CO_) (Fig. 3). Oxygen saturation on air at rest was normal (94–98%) in all patients.

**Fig. 4:**
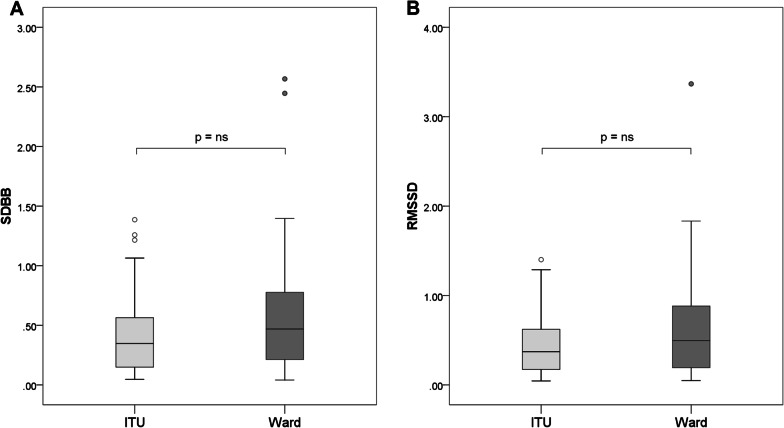
Box and whisker plots comparing breathing entropy measured by SLP in COVID-19 survivors. Neither comparison between ward and ITU patients was statistically different. Entropy was calculated as (**A**) Standard Deviation of the Breath by Breath interval (SDBB) and (**B**) Root Mean Square Standard Deviation (RMSSD). Patients include those who were treated on ITU (light grey boxes, n = 34) and those who were treated on the ward alone (dark grey boxes, n = 45). Boxes represent the IQR with the median line displayed. Whiskers represent 1.5 × IQR to highlight outliers

Some COVID-19 survivors who performed SLP showed abnormality in duty cycle, phase angle and respiratory rate (21.8%, 22.1% and 19.5% abnormality, respectively) compared with reference values. However, there were no statistical differences in SLP measurements between the patients from ITU and those from the ward, including assessments of entropy (Fig. 4).

## Discussion

Our data concur with other lung function reports of COVID-19 survivors [2–5] by showing a restrictive pattern with a reduction in overall gas transfer (TL_CO_) in a proportion of COVID-19 survivors. Pulmonary fibrosis is a recognised sequela of both Adult Respiratory Distress Syndrome (ARDS) and barotrauma due to high pressure mechanical ventilation and may be a contributing factor on COVID-19 patients treated on ITU and, possibly, those treated with high CPAP pressures on the ward (although very few patients received CPAP at University Hospitals Birmingham during the first COVID-19 wave). However, we also note the relatively preserved or slightly raised K_CO_ in 78.1% of these patients, which is usually more consistent with an extrapulmonary pathology. Our data suggest that this pattern of physiological dysfunction may be even more pronounced in COVID-19 patients admitted to ITU who were treated on the ward alone (and weren’t intubated or mechanically ventilated). This pattern was also reported by Mo et al. [2] but was not sufficiently explained. We have considered several possible explanations for this pattern including, Intussusceptive angiogenesis (remnants of lung vascular changes from COVID-19), pulmonary haemosiderosis, and extrapulmonary restriction (obesity, pleural issues or muscle weakness).

The most common cause of this pattern is extrapulmonary restriction, with obesity being the most likely cause [18]. Indeed given the body habitus of all our patients to be predominantly overweight/obese, this seems a likely hypothesis. However, reviewing the literature suggests that this pattern is usually only observed in severe obesity (BMI > 40) [19]. Only 4 ward and 9 ITU patients were above this threshold, so excessive obesity is unlikely to be the sole reason for this pattern. Although extrapulmonary restriction is more associated with upper body fat [20], we didn’t collect this data in our patients.

Several studies suggest that the reduction in volume caused by obesity was insufficient to explain the increase of K_CO_ found in patients with small lung volumes [18, 21]. Usually, TL_CO_ in healthy subjects decreases as the inspired volume reduces at a rate of about 3.3% per 10% decrease of vital capacity. In this current study, the inspired volume (Vi) was 94.8% (3.6%) of the vital capacity (VC) indicating a close match of Vi with VC. On average, VC was reduced by about 15% which would mean Vi should have been reduced by about 5%, but it was reduced by around 15% which suggests more than an obesity effect. It is understood that there is a relative increase in capillary blood volume in obesity that is thought to lead to the increase in gas transfer [22].

Whilst the COVID-19 survivors in the Mo et al. [2] study also showed the same reduced TL_CO_ with raised K_CO_ pattern, their population all had a mean BMI below 25, which suggests that the phenomenon is not related to obesity causing extrapulmonary restriction.

Muscle weakness causing extrapulmonary restriction would show a reduction in muscle pressures. Intensive Care Unit Acquired Weakness (ICUAW) is a well-recognised phenomenon that could potentially cause weakness of the respiratory pump. Logically, this would be more prevalent in COVID-19 survivors treated on ITU but may also be a contributing factor for patients treated on wards who remained virtually bed-bound for long durations. Although we didn’t measure respiratory muscle function, Huang et al. recently showed normal maximal inspiratory/expiratory muscle pressures in post-COVID-19 survivors [3], which argues against this hypothesis. There is no suspicion from the recent COVID-19 literature that respiratory muscle weakness is a feature of COVID-19 recovery, although neuropathy and general fatigue have been noted as a key symptom in sick and recovering patients.

A high K_CO_ indicates a predominance of pulmonary capillary volume (Vc’) over alveolar volume, which may arise for different reasons. Incomplete alveolar expansion but preserved gas exchange unities frequently lead to a K_CO_ of 120–140% predicted or even higher (i.e. extra-parenchymal restriction, such as pleural, chest wall or neuromuscular disease) [21–24]. Pleural and interstitial changes have been identified in COVID-19 patients using imaging [25–29] and at autopsy [30]. Alternatively, an increase in pulmonary blood flow from areas of diffuse (pneumonectomy) or localised (local destructive lesions/atelectasis) loss of gas exchange units to areas with preserved parenchyma can lead to more modest increases in K_CO_. However, a high K_CO_ can also be seen with normal or near-normal V_A_ when there is increased pulmonary blood flow or redistribution (e.g. a left-to-right shunt or asthma). Intussusceptive angiogenesis [31] as a result of chronic infection is a dynamic intravascular process that can modify the structure of the microcirculation. Recently, this has been shown to be present at autopsy in COVID-19 patients [32] and could explain some or most of the rise in K_CO_ observed.

Haemosiderosis, which is the deposition of extra-vascular haemoglobin, may also be a cause. Alveolar haemorrhage is a possible mechanism given the vascular destruction reported in active COVID-19 and fits with the lung function results. In haemosiderosis, macrophages convert the iron in haemoglobin into haemosiderin within 36–72 h [33, 34] and the haemosiderin-laden macrophages can reside for up to 4–8 weeks in the lungs. Pulmonary haemosiderosis is usually considered to be from persistent or recurrent intra-alveolar bleeding, which may explain the symptoms of “long COVID” and the time course of improvement in symptoms. The effect of haemosiderosis on interpretation of the gas transfer test has been highlighted by Hughes [18, 35]. However, there was only mild anaemia (haemoglobin < 120 g/L) in 7 (14.9%) of the ward patients and 12 (25.5%) of ITU patients and all gas transfer tests were corrected for haemoglobin. This makes haemosiderosis an unlikely explanation for most of the abnormalities we observed in gas transfer.

Another explanation for the reduced TL_CO_/raised K_CO_ pattern could be the development of necrotising pulmonary capillaritis occurring in isolation [36]. This arises from diffuse interstitial neutrophilic infiltration with cell fragmentation and, because of apoptosis, cellular accumulation within the lung tissue, filling the interstitial space. This can lead to expansion and fibrinoid necrosis. As a result of these processes, the integrity of interstitial capillaries is damaged, allowing red blood cells to pass through the alveolar capillary basement membranes, freely enter the interstitial compartment and flood alveolar spaces. Clinically, this diffuse alveolar microhaemorrhage enables the CO in the gas transfer test to combine with this “occult” blood or haem from haemosiderosis and effectively raise the K_CO_. However, global gas transfer (TL_CO_) is not as affected because of the counteractive restrictive defect that causes a decrease in lung volume and, hence, alveolar surface area which, in turn, has a greater effect on decreasing the TL_CO_ than the rise due to the diffuse local haemorrhage.

A similar TL_CO_ and K_CO_ pattern seen in SARS [37] was thought to be the result of muscle wasting and corticosteroid induced myopathy. We have insufficient data to prove or disprove this hypothesis currently so, even though it is unlikely, it cannot be excluded.

More males than females (as reported elsewhere in COVID-19) required assisted ventilation/oxygenation on ITU and, therefore, probably had worse infections. These hospitalised patients were predominantly overweight /obese, which is another known risk factor in severe COVID-19 for an increased likelihood of hospitalisation, ITU admission and morbidity. Ethnicity is also known to be a factor associated with an increase in incidence and severity of COVID-19 in patients from black, Asian and minority ethnic (BAME) communities in the UK [38]. However, we did not note any significant differences in ethnicity between ITU and ward patients, so this does not appear to be causal in the outcome of the gas transfer tests.

There were no major differences in lung function between ward and ITU patients that could clearly differentiate ward and ITU patients, despite a statistically lower K_CO_ on average in ITU patients (where there was much overlap between the two cohorts). It might have been expected that patients who had had mechanical ventilation for severe COVID pneumonitis to have had a more obvious and distinct pattern of lung function impairment but this wasn’t the case at 3+ months.

Our data also show that few of the patients had any evidence of airflow obstruction on spirometry (FEV_1_/FVC SR) and that never-smokers showed greater hospitalisation but this may be because smokers (more likely to have COPD) either shielded from COVID-19 and never got the infection, or died on ITU and weren’t followed up. Some may interpret this as evidence of the “protective effect” of smoking in COVID-19 [39].

### Breathing patterns

The anticipated alteration in breathing patterns was not evident when compared with reference values. The ITU patients had no more dysfunctional breathing patterns than the ward patients. Whilst many post-ITU patients display dysfunctional breathing immediately on leaving ITU, it appears to improve rapidly in most, so by 3+ months there are only 20% showing abnormality.

These abnormal SLP values were both lower and greater than the normal range with no consistent pattern. We had wondered whether SLP could have been used to detect dysfunctional breathing patterns in COVID-19 survivors, linked to the severity of impaired gas transfer and, therefore, lung damage. However, this relationship wasn’t strong, so screening for lung function impairment should continue to use traditional spirometry and gas transfer in patients who have symptoms compatible with post-COVID-19 lung changes.

The reasons for abnormal breathing patterns could be the result of (a) obesity, (b) COVID-19 itself causing pneumonia, leading to sepsis, and producing delirium, (c) the effects of sedation and medication on breathing centres, or (d) mechanical ventilation and oxygen therapy.

It is well established that the work of breathing is increased and the total respiratory compliance is decreased in obesity [19]. This could be a cause of altered breathing patterns, although there is no obvious link between the two in this data. Further work is being undertaken to explore these breathing patterns in more detail.

## Limitations

The potential errors with lung function testing have been minimised since all testing was performed on calibrated equipment and was measured by experienced, well-trained personnel. In addition, all equipment was monitored with a stringent quality control protocol, including both physical and biological quality control, within tests quality checks and review of all tests by senior physiologists [7, 8]. Furthermore the V_A_/TLC ratio in the sub group who had lung volumes measured showed V_A_ to be on average within 7% of TLC, which indicates good consistency and test quality. Unfortunately, we were unable to perform lung volume measurements in all patients due to limited lung function timeslots.

Our population and their treatment may be different from other centres who have published lung function data in COVID-19. Certainly the body habitus of the data from Mo et al. [2] shows normal BMI values, unlike our population who were predominantly obese/overweight. However, the ventilation regimens adopted in the UK and at our centre were based around the WHO guidance for COVID-19 following experience from Wuhan early in the pandemic [1].

We didn’t measure muscle pressures as this wasn’t a prospective study. However, Huang et al. [3] found no abnormalities in respiratory muscle function. We also didn’t measure abdominal obesity as this may have a different effect on gas transfer compared with upper body obesity.

Changes we have seen may not just be due to COVID-19 directly but, also, therapeutic insults/interactions (e.g. corticosteroids, oxygen, and mechanical ventilation) or other pathophysiological events such as delirium or sepsis. However, similar regimens were used across world after the Wuhan experience was published. Nevertheless, the physiological changes in this population will remain a legacy for many patients who have had COVID-19 and will add further demands to already over-subscribed, limited in performance lung function facilities worldwide [40]. Consequently, because of aerosol-generating properties of lung function testing and the difficulties delivering testing [40], it has not been possible to test all patients at the same time since hospital discharge.

The reference values for breathing patterns (using SLP) are a recently derived and validated set of references values and may not be good at discriminating normal from abnormal.

In summary, we have been able to identify common pattern of restrictive pulmonary impairment in COVID-19 recovery but not the specific causes. There are many potential contributing factors both directly related to the infection but, also, other patient-specific factors such as co-morbidities, treatment regimes, length of stay, physical fitness, etc. In addition, radiological data and symptom scores were not available. Due to the multifaceted nature of the clinical outcome in COVID-19, it would most likely require a much larger population with additional information to elicit the specific pathologies that induce these physiological effects.

### Future work

Future work should measure TL_NO_ or Dm/Vc’ in COVID-19 so that the vascular component and membrane components of the gas transfer processes can be better understood. It would be expected that a pattern consistent with altered capillary blood volume may become evident.

Some patients in our post-COVID-19 clinics will have further follow-up lung function after another 3+ months, so it will be interesting to see if the changes we have found (particularly in gas transfer) are related to any change in body habitus or to the repair of the suspected lung damage we have highlighted here. Further analysis of the breathing pattern data may show subtle differences in breathing patterns between the two groups.

## Summary

We found similar restrictive patterns (reduced vital capacity and alveolar volume) in survivors with moderate and severe COVID pneumonitis whether admitted to wards or ITU. There is often a mild reduction in gas transfer (TL_CO_) but a preservation/ relative rise in transfer coefficient (K_CO_). These results can be explained partly by obesity (causing extrapulmonary restriction) but perhaps also by haemosiderosis from lung damage and localised microvascular changes in lung capillaries. Potential respiratory muscle fatigue/weakness is unlikely to be a causal factor in our study.

Abnormal breathing patterns (outside 1.64 SRs) of reference data showed 20% of subjects displayed one or more abnormality of breathing in duty cycle, phase angle and respiratory rate. However, no consistent breathing pattern abnormalities were evident. The use of common breathing pattern indices to screen post-COVID-19 patients for those who require more extensive lung function testing isn’t borne out in our population.

## Conclusion

We conclude that the residual changes in lung function and breathing patterns observed at 3+ months are similar whether patients attended wards or were mechanically ventilated on ITU, despite a minor (albeit statistically significant) difference in K_CO_. Understanding the patterns of physiological abnormality post-COVID-19 could help direct clinical management of these patients during their recovery.

## Data Availability

The datasets during and/or analysed during the current study available from the corresponding author on reasonable request.

## Abbreviations

BMI: Body Mass Index
COPD: Chronic Obstructive Pulmonary Disease
COVID-19: Coronavirus Disease 2019
CPAP: Continuous Positive Airway Pressure
Dm: Membrane Diffusing Capacity
ECSC: European Coal and Steel Community
FEV1: Forced Expired Volume in 1 Second
FiO_2_: Fraction of Inspired Oxygen
FVC: Forced Vital Capacity
GLI: Global Lung Initiative
ICUAW: Intensive Care Unit Acquired Weakness
IE50: Inspiratory:Expiratory Flow Ratio at 50% Tidal Volume
IQR: Interquartile Range
ITU: Intensive Therapy Unit
K_CO_: Transfer Coefficient for Carbon Monoxide
MERS: Middle East Respiratory Syndrome
NIV: Non-Invasive Ventilation
PA: Phase Angle
PEF: Peak Expiratory Flow
RMSSD: Root Mean Square of the Successive Differences between Each Breath
RR: Respiratory Rate
RTC: Relative Thoracic Contribution
SARS-CoV-2: Severe Acute Respiratory Syndrome Coronavirus 2
SDBB: Standard Deviation of Breath to Breath
SE: Standard Error of the Mean
SLP: Structured Light Plethysmography
SR: Standardised Residuals
Ti/Ttot: Inspiratory Time/Total Breath Time (Duty Cycle)
TLC: Total Lung Capacity
TL_CO_: Transfer Factor for Carbon Monoxide
TL_NO_: Transfer Factor for Nitric Oxide
V_A_: Alveolar Volume
VC: Vital Capacity (Relaxed)
Vc’: Pulmonary Capillary Volume
Vi: Inspired Volume
